# 

**Published:** 1909-08-15

**Authors:** 


CONTENTS

Event and Comment - - - - - - - - 389

Early Care of Children's Teeth, with Special Reference to the Prevention of Facial and Oral Deformities,
*By Milton T. Watson, D.D.S.* 390

The Diseases of the Pulp, - *By Russell W. Bunting, D.D.Sc.* 411

Humane Methods of Wedging,
*By S. E. Davenport, D.D.S., M.D.S.* 416

Bi-Fluoride of Ammonium, - - *By Joseph Head, D.D.S.* 422

Indents - - - - - - - - - - - - 430

Announcements - - - - - - - - - - 440

